# The relationships between the medical learners’ motivations and strategies to learning medicine and learning outcomes

**DOI:** 10.1080/10872981.2018.1497373

**Published:** 2018-07-16

**Authors:** Jyh-Chong Liang, Yen-Yuan Chen, Hong-Yuan Hsu, Tzong-Shinn Chu, Chin-Chung Tsai

**Affiliations:** a Program of Learning Sciences, National Taiwan Normal University, Taipei, Taiwan; b Institute for Research Excellence in Learning Sciences, National Taiwan Normal University, Taipei, Taiwan; c Department of Medical Education, National Taiwan University Hospital, Taipei, Taiwan; d Graduate Institute of Medical Education & Bioethics, National Taiwan University College of Medicine, Taipei, Taiwan

**Keywords:** The approach to learning, learning outcome, surface strategy, surface motivation, deep strategy, deep motivation

## Abstract

**Background**: One of the strongly theorized areas of research associated with learning outcomes has been the approaches to learning. Few studies have been focused on examining the relationship between the approaches to learning medicine (ALM) and learning outcomes.

**Objectives**: The objectives were: (1) to conduct psychometric testing of the ALM questionnaire; and (2) to examine the association between medical learners’ ALM and learning outcomes.

**Design**: We developed the ALM questionnaire which was a modification of the Revised Learning Process questionnaire. We defined the learning outcome of each house officer as the class rank in his/her graduating class. Exploratory factor analysis was used to examine the factor structure of the ALM questionnaire. We conducted Pearson’s and Spearman’s Rank correlation coefficients for examining the linear relationships between two continuous variables, and between a continuous variable and a categorical variable, respectively. Stepwise multivariate linear regression analysis with backward elimination was undertaken to examine the correlation between the ALM and the learning outcome.

**Results**: A house officer with deep strategies (relating multiple ideas and truly understanding the course content) or surface motivations (aim for qualification) was more likely to have a better learning outcome as indicated by a better class rank based on his/her academic performance. Furthermore, a house officer with surface learning strategies (minimizing the study scope to merely passing the examination) to learning medicine was more likely to have an unfavorable class rank.

**Conclusions**: This study represents the first report of the correlation between house officers’ ALM and learning outcomes. House officers with deep strategies were more likely to have better learning outcomes. In particular, house officers with a surface motive to learning medicine, i.e., aiming for qualification, were surprisingly correlated with better learning outcomes.

## Introduction

Medical students’ learning outcomes have been of great importance both for medical students and for medical educators. Medical educators are concerned with learning outcomes because they are expected to cultivate well-trained medical students in response to the needs of the society and healthcare systems. Medical students are also concerned with their learning outcomes because learning outcomes are so closely related to their future application to residency training, i.e., the specialty they are able to select and the healthcare institution where they will receive residency training.

One of the more strongly theorized areas of research associated with learning outcomes has been students’ approaches to learning. ‘Approaches to learning’ implies the learners’ motivations and strategies to learn or process academic work [1]. Prior studies have reported two major approaches to learning: surface approaches and deep approaches [2,3]. The surface approaches to learning (surface strategies) are built on external interests, or extrinsic motivations, indicating that students learn by rote through repeatedly rehearsing or memorizing. The utilization of surface approaches to learning may involve the fear of failure, the intention of merely passing the exams, or the effort just to carry out the learning tasks. In comparison, the deep approaches to learning (deep strategies) are built on internal interests, or intrinsic motivation in the learning process, indicating that students learn by aiming at understanding the learning content, or a more coordinated way of learning such as connecting what one already knows or has learned to the current course content [2,4]. Currently, the distinctions between the two strategies, as well as the two motivations, have been studied in learning sciences [5] and in learning biology [4].

Research has continuously reported that students’ approaches to learning are associated with learning outcomes [6–8]. Snelgrove and colleagues reported that student nurses’ deep approaches to learning sociology were positively associated with their learning outcome as indicated by grade point average (GPA) and sociology exam results [9]. Hassall and colleagues reported that the professional accounting students with deep approaches to learning scored higher than those with surface approaches to learning in the qualification examination [10]. Chamorro-Premuzic and colleagues also reported that the deep approaches to learning accounted for more of the variances in students’ academic performance as indicated by the grades in exams than those accounted for by their personality and intelligence [11]. Most of the previous research on examining the association between the approaches to learning and learning outcomes used students’ GPA or grades in exams as the measurement of learning outcomes.

Although factors associated with the approaches to learning have been well examined in scientific and in non-medical learners [4,5,12,13], few studies in medical education have been focused on examining the issues associated with the approaches to learning medicine (ALM), and used class rank as a measurement of the learning outcome. The objectives of this study were: (1) to conduct a factor analysis and further psychometric testing of the newly developed ALM questionnaire; and (2) to examine the association between medical learners’ ALM and the learning outcomes.

## Methods

### Study design

We conducted this study in a university-affiliated medical center located in northern Taiwan. A total of 126 post-graduate year 1 (PGY) house officers who received general medical training from 2015 to 2016 in this medical center were eligible for this study. At the beginning of their training, we inquired about the 126 house officers’ willingness to join this study. Questionnaires were then distributed to the house officers who agreed to join.

### Instrument

To thoroughly assess house officers’ ALM, the ALM questionnaire was developed which was a modification of the previous questionnaire, Revised Learning Process Questionnaire (R-LPQ-2F), established by Biggs and colleagues [14]. The R-LPQ-2F had been confirmed through confirmatory factor analysis consisting of eight factors with deep approaches to learning (goodness of fit indexes: CFI = 0.969 and SRMR = 0.027) including deep motivations and deep strategies, and surface approaches to learning (goodness of fit indexes: CFI = 0.965 and SRMR = 0.024) including surface motivations and surface strategies. The eight factors in four subscales in their study included: ‘Intrinsic Interest’ and ‘Commitment to Work’ as deep motivations; ‘Relating Ideas’ and ‘Understanding’ as deep strategies; ‘Fear of Failure’ and ‘Aim for Qualification’ as surface motivations; and ‘Minimizing Scope of Study’ and ‘Memorization’ as surface strategies.

In order to examine students’ domain-specific approaches to learning (e.g., learning science, biology, medicine, and so on), several studies have modified and re-validated R-LPQ-2F questionnaire [4,15]. For the purpose of this study, the R-LPQ-2F was adopted and modified, in an attempt to examine house officers' approaches to learning medicine. We took the following steps to generate the ALM questionnaire based on the R-LPQ-2F:

Firstly, we changed the learning context in the R-LPQ-2F to the learning context of ‘learning medicine.’ For example, the original item ‘I find that at times studying makes me feel really happy and satisfied’ in the R-LPQ-2F was changed to ‘When learning medicine, I find that at times studying medicine makes me feel really happy and satisfied’ in the ALM questionnaire.

Secondly, we added and deleted some items for measuring house officers’ ALM based on the discussions with house officers. For example, one item ‘I find the best way to pass examinations is to try to remember answers to likely questions’ in the ‘Memorization’ factor was suggested to be deleted because of the unsuitability. In addition, one more item ‘When learning medicine, I use systemic ways to learn’ in the ‘Understanding’ factor was suggested and then added in the final version.

Thirdly, some house officers were invited to examine the clarity and understandability of all the items shown in the ALM questionnaire. Further minor revisions for the wording of certain items were made based on their suggestions and comments.

Fourthly, a higher education and a medical education expert evaluated all of the 36 questionnaire items and approved the use of the ALM questionnaire. The four subscales and eight factors with sample items are presented below:

Deep Motivations: The medical learners have deep motives (i.e., having their own intrinsic interest and work commitments) to learning medicine.

Factor 1: Intrinsic Interest

 Sample item: When learning medicine, I find that at times studying medicine

makes me feel really happy and satisfied.

Factor 2: Commitment to Work

 Sample item: In my leisure time, I work on the medical issues which I am

interested in, and which have been discussed in medical fields.

Deep Strategies: The medical learners tend to use deep strategies (i.e., relating multiple or previous ideas and truly understanding the content of the text books) to learning medicine.

Factor 1: Relating Ideas

Sample item: When learning medicine, I try to relate what I have learned in one

subject to what I have learned in others.

Factor 2: Understanding

Sample item: When reading medical textbooks, I try to understand the meaning of

the content.

Surface Motivations: The medical learners have surface motives (i.e., fear of failure of the examinations or just aiming for qualification) to learning medicine.

Factor 1: Fear of failure

Sample item: I am worried about my performance in future National Licensing

Exam if I get bad grades in current exams.

Factor 2: Aim for Qualification

Sample item: Regardless of whether I like it or not, I know that having good

performance in medicine can help me to find a good job in the future.

Surface Strategies: The medical learners tend to use surface strategies (i.e., minimizing the study scope to pass the examinations or just using rote learning) to learning medicine.

Factor 1: Minimizing Scope of Study

Sample item: When learning medicine, the items not tested in the exams are

meaningless to me.

Factor 2: Memorization

Sample item: When learning medicine, I practice rote memorization of the content

until I firmly memorize all of the content.

Each factor contained a total of three to seven items. We coded all the items using a Likert scale ranging from 1 to 5, indicating ‘strongly disagree’ to ‘strongly agree,’ respectively. The score of each factor for a house officer was derived by taking the average of all the items included in the factor.

### Learning outcomes

We defined the learning outcomes of each house officer in this study as the class rank of the house officer in his/her graduating class [16]. It can be calculated by the following algebraic function:

Class Rank = (the total number of medical students with a lower average grade than the house officer in his/her graduating class) / (the total number of medical student in the house officer's graduating class).

For example, someone who is the top student based on her average grade in her graduating class with a total of 100 medical students has a class rank as 0.99 based on the algebraic function. The formula for class rank was purely mathematical – based on the average score derived from all the exams taken, all the ratings given during the 7 years in his/her medical school. The study participants from the same medical schools took the same exams during preclinical years, and mostly the same clinical rotations during clinical years. In addition, medical students from different medical schools were required to take the same courses, and mostly similar clinical rotations by Taiwan Ministry of Education for getting a M.D. degree.

### Statistical analyses

The ALM questionnaire was first developed for assessing a medical learner’s various ALM. Thus, exploratory factor analysis was used to examine the factor structure of the ALM questionnaire, as well as its validity and reliability.

We conducted Pearson’s correlation coefficients and Spearman’s Rank correlation coefficients for examining the linear relationships between two continuous variables (e.g., age and the rank in his/her graduating class), and between a continuous variable and a categorical variable (e.g., gender and the rank in his/her graduating class), respectively.

Stepwise multivariate linear regression with backward elimination was then undertaken in this study. Given the potential influence of age and gender [17], the age upon participation in this study and gender were included as the confounding variables. The ALM with a *p* value of less than or equal to 0.20 were remained in multivariate analysis. The Class Rank in his/her graduating class in medical school was the dependent variable. Adjusted R square was used to examine the model fit.

A two-tailed *p* value of less than or equal to 0.05 was considered as statistically significant. All statistical analyses were conducted using SPSS (Statistical Package for the Social Sciences) 20.0 for Windows PC. This study was approved by the National Taiwan University Social and Behavioral Research Ethics Committee (201505HS002).

## Results

One hundred and twelve (88.9%) of the 126 eligible house officers signed the informed consent form, and finally 107 (84.9%) completed the ALM questionnaire and were included in the data analysis. The age ranged from 24.25 to 32.74, with a mean (±standard deviation) of 26.34 (±1.44) years old. Sixty-seven (62.6%) of the 107 participants were male.

### Factor analysis

To validate the ALM questionnaire, we adopted the principal component analysis with a varimax rotation method to clarify the factors of the items. The eigenvalues of all the factors were greater than one, and a factor loading of greater than or equal to 0.5 was used as the criterion for retention of items. According to the factor analysis of the ALM questionnaire, five items were removed, and ‘Relating Ideas’ and ‘Understanding’ were merged into a single factor called ‘Relating Ideas and Understanding.’ Therefore, seven factors with a total of 31 items, accounting for 68.1% of the total variance, were included for further analyses. In addition, the Cronbach’s alpha values for each factor ranged from 0.66 to 0.88, with an overall value of 0.76, suggesting these seven factors in the ALM questionnaire had adequate internal consistency for assessing house officers’ ALM.

As shown in Table 1, the house officers scored highly on ‘Relating Ideas and Understanding’ for deep learning strategies. In contrast, their scores on the ‘Aim for Qualification’ for surface motivations and ‘Minimizing Scope of Study’ for surface strategies to learning medicine were relatively much lower than the other factors in the ALM questionnaire.

**Table 1.** Rotated factor loadings, Cronbach’s alpha values and mean scores for the seven factors of the ALM questionnaire.

	Item	Factor loading	Cronbach’s alpha	Mean (±SD)
Deep motivations				
II 1	When learning medicine, I find that at times studying medicine makes me feel really happy and satisfied.	0.77	0.88	3.30 (±0.73)
II 2	When learning medicine, I feel that the content is very interesting.	0.78		
II 3	I find myself very engrossed in learning medicine because I am interested in the subject.	0.80		
II 4	I always look forward to medical courses.	0.74		
II 5	I attend medical school because I especially like to study medicine-related issues.	0.64		
CW 1	In my leisure time, I work on the medical issues which I am interested in, and which have been discussed in medical fields.	0.71	0.79	3.47 (±0.61)
CW 2	Before a medical class starts, there are always many unanswered questions on my mind.	0.50		
CW 3	Even outside of the time of my medical class, I still think of the learning content related to the class.	0.68		
CW 4	I feel satisfied that I like to use my medical studies to make conclusions.	0.78		
Deep strategies				
RIU 1	When learning medicine, I try to relate what I have learned in one subject to what I learn in others.	0.67	0.87	3.96 (±0.45)
RIU 2	When learning medicine, I like to create a new plausible theory for helping me to summarize a lot of disorganized content.	0.56		
RIU 3	When learning medicine, I try to find out the relationships between what I have learned.	0.81		
RIU 4	When learning new subjects in medicine, I try to relate to those I have learned.	0.82		
RIU 5	When reading medical textbooks, I try to understand the meaning of the content.	0.80		
RIU 6	When learning medicine, I try to understand the content of the medical courses.	0.68		
RIU 7	When learning medicine, I use systemic ways to learn.	0.64		
Surface motivations				
FF 1	I am worried about my performance in future National Licensing Exams if I get bad grades in current exams.	0.56	0.71	3.45 (±0.78)
FF 2	Although I have tried my best to prepare for the exams, I am still worried about not doing well in the exams.	0.88		
FF 3	I am worried that my academic performance in medical courses does/may not meet the expectation of teachers and family members.	0.65		
AQ 2	I am eager to get good grades in medical courses in expectation of getting a good job offer.	0.67	0.66	3.18 (±0.71)
AQ 3	For making family members and teachers happy, I want to have good academic performance in medical subjects.	0.83		
AQ 4	I work very hard to prepare for the exams in medicine to achieve family members’ expectations to be a physician.	0.65		
Surface strategies				
MSS 2	When learning medicine, I spend as little time on studying medicine as I can, as long as I feel that I can pass the exams. There are many other interesting things to do.	0.68	0.76	2.97 (±0.69)
MSS 3	When learning medicine, I find out the contents which are worth spending time to study simply because I do not want to spend time and energy on the unrelated content.	0.68		
MSS 4	When learning medicine, we do not need to be familiar with all the contents simply because there are so many exams we need to pass, and so many subjects we need to learn.	0.73		
MSS 6	I feel that the best way to get good grades in medical exams is the rote the answers of similar questions.	0.57		
MSS 7	I find that to memorize the content of medical subjects by rote makes me achieve good grades on exams, rather than to understand them.	0.64		
M 1	When learning medicine, rote is the strategy I use until I firmly memorize all of the content.	0.77	0.80	3.68 (±0.63)
M 2	When learning medicine, I am very focused on those which will be tested in exams, and I memorize them by rote.	0.67		
M 3	When learning medicine, I use lecture notes and aids for National Licensing Exams as important learning guides.	0.69		
M 4	When learning medicine, I try to improve my memorization by repeated rote.	0.81		

ALMQ: the Approaches of Learning Medicine Questionnaire; SD: standard deviation; II: intrinsic interest; CW: commitment to work; RIU: relating ideas and understanding; FF: fear of failure; AQ: aim for qualification; MSS: minimizing scope of study; M: memorization.

**Deep Motivations**: ‘intrinsic interest’ and ‘commitment to work’; **Deep Strategies**: ‘relating ideas and understanding’; **Surface Motivations**: ‘fear of failure’ and ‘aim for qualification’; **Surface Strategies**: ‘minimizing scope of study’ and ‘memorization.’

### Correlation coefficients

We found that a house officer’s class rank in his/her graduating class in medical school was positively correlated to intrinsic interest (*r* = 0.29, *p *< 0.01), relating ideas and understanding (*r* = 0.35, *p *< 0.01), and aim for qualification (*r* = 0.22, *p *= 0.03). That is, the house officers with deep motivations (such as intrinsic interest) and deep strategies (such as relating prior knowledge or truly understanding the content) were more likely to have a better learning outcome as indicated by their class rank in his/her graduating class. In addition, house officers’ surface motivations (such as aiming for qualifying for the National Licensing Exam) likely fostered their academic performance in the class (Table 2).

In comparison, house officers’ class rank was negatively correlated to male gender (*r* = −0.19, *p *= 0.05) and minimizing scope of study (*r* = −0.24, *p *= 0.01), the surface strategy of the ALM. That is, house officers with surface strategies of the ALM were more likely to have a poorer learning outcome as indicated by their class rank in his/her graduating class. In addition, male house officers had a lower class rank than female house officers.

### The ALM and learning outcomes

Based on the correlation results, we further conducted stepwise multivariate linear regression with backward elimination to predict house officers’ learning outcome as indicated by class rank. The regression analysis showed that a house officer’s age and gender were not significantly associated with the class rank. In comparison, a house officer’s ALM i.e., ‘Relating Ideas and Understanding’ (deep strategy) and also ‘Aim for Qualification’ (surface motivation) significantly predicted his/her higher class rank. In addition, a house officer’s ALM, i.e., ‘Minimizing Scope of Study’ (surface strategy) significantly predicted his/her lower class rank (Table 3).

**Table 2.** The correlations coefficients between the medical learners’ class rank, age, gender and the seven factors in the ALM questionnaire (*n* = 107).

	Deep motivations		Deep strategies	Surface motivations		Surface strategies		
	II	CW	RIU	FF	AQ	MSS	M	Class rank
Class rank	0.29^#^	0.18^+^	0.35^#^	0.01	0.22*	−0.24*	−0.10	
Age	−0.02	−0.07	−0.06	0.08	−0.03	0.12	−0.05	−0.03
Gender (0: female)	−0.08	−0.05	−0.11	−0.21*	−0.26^#^	0.20*	0.02	−0.21*

II: intrinsic interest; CW: commitment to work; RIU: relating ideas and understanding; FF: fear of failure; AQ: aim for qualification; MSS: minimizing scope of study; M: memorization. Notes: + *p* < 0.20, *p < 0.05, #*p* < 0.01.

**Table 3.** Stepwise multivariate linear regression model of predicting the learning outcome (*n* = 107).

	Coefficient	*p*-Value
Relating ideas and understanding (deep strategy)	0.14	<0.01
Aim for qualification (surface motivation)	0.08	0.01
Minimizing scope of study (surface strategy)	−0.07	0.03

Adjusted R-squared = 0.17.

## Discussion

### Main findings

The results showed that house officers’ ALM played important roles in their academic performance. A house officer with deep strategies (i.e., relating multiple ideas and truly understanding the course content) or surface motivations (i.e., aim for qualification) was more likely to have a better learning outcome, i.e., a higher class rank based on his/her academic performance. Moreover, a house officer with surface learning strategies (i.e., minimizing the study scope to merely passing the examination) to learning medicine was more likely to have an unfavorable learning outcome as indicated by his/her class rank.

### Deep ALM and learning outcomes

Two studies have reported that deep approaches to learning were dominant among the learners regardless of their gender and age [18,19]. Deep approaches to learning have been recognized to be associated with the quality of the learning outcome [6]. Most studies have continuously shown that deep motivations and deep strategies of learning are associated with better learning outcomes [9]. In this study, deep strategies have also been shown to be associated with better learning outcomes. As indicated by deep strategies, our study results based on medical learners were the same as those based on non-medical learners.

However, deep motivations, i.e., ‘Intrinsic Interest’ and ‘Commitment to Work,’ did not predict house officers’ learning outcomes. This finding seemed to be inconsistent to prior study results [9]. The study design may be attributed to this finding. We measured the study house officers’ ALM right after they completed learning in medical school, and assumed it was unchanged. Actually, medical students, while entering medical school, may have intrinsic interests and are also committed to their work to learn medicine. However, after several years of exhausting learning, they may not have had intrinsic interests and commitment upon graduation when we measured their ALM.

### Surface ALM and learning outcomes

Several studies have shown that learners with surface approaches to learning have had a negative significant impact on their academic performance. Consistent with prior studies, we found that surface strategies (i.e., minimizing the scope of studying medicine) were negatively associated with the learning outcome. Interestingly, we also found that a house officer with surface motivations, i.e., aiming for qualification, was associated with a better learning outcome. This finding based on medical learners was in opposition to those reported based on non-medical learners. Several reasons may account for the finding we identified in this study:

Firstly, medical learners are very sophisticated in their ALM based on different motivations. They may have adaptive motivations, i.e., changing from deep motivations to surface motivations, or from surface motivations to deep motivations, depending on many other factors such as learning content, learning context, and availability of time and effort. For example, if a medical learner does not have much time for learning a medical subject, his/her motivation to learn the medical subject may be simply to pass the exam or the fear of failing the exam. Based on the two surface motivations, he/she can still be very good at learning the medical subject and attaining good grades. In comparison, if a medical learner has plenty of time for learning a medical subject, he/she may be encouraged to learn by deep motivations, and still maintain quality learning and earn satisfactory grades. In addition, they may possess deep motivations and surface motivations simultaneously to learning medicine. Chiu et al. reported that Taiwanese medical students with epistemic beliefs related to justifying medical knowledge were more likely to have mixed motivations to learning medicine [20].

Secondly, ‘Aim for Qualification’ to house officers is not a good indicator for the surface motivation of the ALM. The most important difference between medical learners and non-medical learners may be how they perceive ‘Qualification.’ Getting accepted into a medical school is so competitive that those who seek entry may start to see each exam as a qualification. In medical schools, not only the results of the National Licensing Exam, but also the grade of each course are closely related to a medical students’ future career. In comparison, non-medical learners may not need to take so many exams, and the results of each exam may not be as related to or important for their future career. Therefore, ‘Aim for Qualification’ to medical learners, unlike non-medical learners, may not be a good indicator of surface motivation.

Our study reported that ‘fear of failure’ and ‘memorization’ did not predict medical students’ learning outcomes. The results are not surprising because of the characteristics of the study participants. Our study participants were the house officers with teaching and learning in clinical training years. Therefore, they were not so stressful about pass/fail issues, and rote memorization of countless details of basic medical sciences as compared to the medical students in the preclinical years.

### Strengths and limitations

We examined the association between house officers’ ALM and their learning outcomes as indicated by the class rank in his/her graduating class in medical school, which has not been used in studies to examine the association between the ALM and the learning outcomes. In addition, the participants from several different medical schools and the high participation rate guaranteed a relatively satisfactory external validity. Nevertheless, there were several limitations in this study.

Firstly, this was a single-center study. The health care institution where this study was conducted was one of the best medical centers for training house officers. Thus, the house officers accepted for training in this health care institution might represent those with better academic performance. This might limit the generalizability of our study results.

Secondly, it is possible that potential confounding variables were not included in the multivariate linear regression model. Although we already included personal characteristics, i.e., age and gender, as the independent variables in the model, there is always the chance that some unknown potential confounding variables were not collected.

Thirdly, the questionnaire was not anonymous, and that might influence participants’ willingness to report their learning strategies and motivations truly.

## Conclusion

This study first showed that a house officer’s ALM, such as deep motivations, deep strategies, surface motivations, and surface strategies, significantly predicted the learning outcome. In particular, a house officer with a surface motive to learn medicine, i.e., aiming for qualification, surprisingly predicted a higher class rank in his/her graduating class. Although several possibilities have been proposed to account for this phenomenon, future studies are highly suggested to further explore this result which was different from that has been reported in the literature.

## Appendix

**Table:** **Appendix 1**. The ALM questionnaire.

	No.	Items
II 1	1	When learning medicine, I find that at times studying medicine makes me feel really happy and satisfied.
II 2	2	When learning medicine, I feel that the content is very interesting.
II 3	3	I find myself very engrossed in learning medicine because I am interested in the subject.
II 4	4	I always look forward to medical courses.
CW 1	5	In my leisure time, I work on the medical issues which I am interested in, and which have been discussed in medical fields.
CW 2	6	Before a medical class starts, there are always many unanswered questions on my mind.
CW 3	7	Even outside of the time of my medical class, I still think of the learning content related to the class.
CW 4	8	I feel satisfied that I like to use my medical studies to make conclusions.
II 5	9	I attend medical school because I especially like to study medicine-related issues.
CW 5	10	I hope to be a physician because I am vivacious, and like to approach people and help them.
RI 1	11	When learning medicine, I try to relate what I have learned in one subject to what I have learned in others.
RI 2	12	When learning medicine, I like to create a new plausible theory for helping me to summarize a lot of disorganized content.
RI 3	13	When learning medicine, I try to find out the relationships between what I have learned.
RI 4	14	When learning new subjects in medicine, I try to relate to those I have learned.
U 1	15	When reading medical textbooks, I try to understand the meaning of the content.
U 2	16	When learning medicine, I try to understand the content of the medical courses.
U 3	17	When learning medicine, I use systemic ways to learn.
U 4	18	When learning medicine, I read original textbooks or use online resources for better understanding of the content.
FF 1	19	I am worried about my performance in future National Licensing Exams if I get bad grades in current exams.
FF 2	20	Although I have tried my best to prepare for the exams, I am still worried about not doing well in the exams.
FF 3	21	I am worried that my academic performance in medical courses does/may not meet the expectation of teachers and family members.
AQ 1	22	Regardless of whether I like it or not, I know that having good performance in medicine can help me to find a good job in the future.
AQ 2	23	I am eager to get good grades in medical courses in expectation of getting a good job offer.
AQ 3	24	For making family members and teachers happy, I want to have good academic performance in medical subjects.
AQ 4	25	I work very hard to prepare for the exams in medicine to achieve family members’ expectations to be a physician.
MSS 1	26	When learning medicine, the items not tested in the exams are meaningless to me.
MSS 2	27	When learning medicine, I spend as little time on studying medicine as I can, as long as I feel that I can pass the exams. There are many other interesting things to do.
MSS 3	28	When learning medicine, I find out the contents which are worth spending time to study simply because I do not want to spend time and energy on the unrelated content.
MSS 4	29	When learning medicine, we do not need to be familiar with all the contents simply because there are so many exams we need to pass, and so many subjects we need to learn.
MSS 5	30	When learning medicine, I hope that the teacher can give us what will be tested in the exams, for us to better prepare for the exams.
M 1	31	When learning medicine, I practice rote memorization of the content until I firmly memorize all of the content.
MSS 6	32	I feel that the best way to get good grades in medical exams is the rote the answers of similar questions.
MSS 7	33	I find that to memorize the content of medical subjects by rote makes me achieve good grades on exams, rather than to understand them.
M 2	34	When learning medicine, I am very focused on those which will be tested in exams, and I memorize them by rote.
M 3	35	When learning medicine, I use lecture notes and aids for National Licensing Exams as important learning guides.
M 4	36	When learning medicine, I try to improve my memorization by repeated rote.

II: intrinsic interest; CW: commitment to work; RI: relating ideas; U: understanding; FF: fear of failure; AQ: aim for qualification; MSS: minimizing scope of study; M: memorization.
